# Speed-dependent modulations of asymmetric center of body mass trajectory in the gait of above-knee amputee subjects

**DOI:** 10.3389/fspor.2023.1304141

**Published:** 2024-01-04

**Authors:** Ken Takiyama, Hikaru Yokoyama

**Affiliations:** ^1^ Department of Electrical Engineering and Computer Science, Tokyo University of Agriculture and Technology, Koganei, Japan; ^2^ Division of Advanced Health Science, Tokyo University of Agriculture and Technology, Koganei, Japan

**Keywords:** Fourier series expansion, dynamic stability, margin of stability (MoS), microprocessor-controlled knee, extrapolated center of mass

## Abstract

How to achieve stable locomotion while overcoming various instabilities is an ongoing research topic. One essential factor for achieving a stable gait is controlling the center of body mass (CoM). The CoM yields more instability in the mediolateral direction. Examining speed-dependent modulations of the CoM trajectories in the frontal plane can provide insight into control policies for achieving stable locomotion. Although these modulations have been studied while assuming symmetric CoM trajectories, this assumption is generally incorrect. For example, amputee subjects demonstrate asymmetric CoM trajectories. Here, we investigated speed-dependent modulations of asymmetric CoM trajectories in above-knee amputee subjects using Fourier series expansion. Despite the asymmetric CoM trajectories in amputee subjects, the framework of Fourier series expansion clarified that amputee subjects showed the same speed-dependent modulations as non-amputee subjects whose CoM trajectories were symmetric. Specifically, CoM trajectories became narrower in the mediolateral direction and broader in the superoinferior direction as walking speed increased. The speed-dependent modulations of CoM trajectories had a functional role in improving dynamic stability, and faster walking speeds provided greater dynamic stability on both prosthetic and non-prosthetic sides. Although the asymmetry of foot contact duration and CoM trajectory decreased as walking speed increased, step width and the asymmetry of dynamic stability between prosthetic and non-prosthetic sides remained constant across the walking speed, which corresponded to the predictions by our framework. These findings could offer a better strategy for achieving stable walking for amputee subjects.

## Introduction

Our bodies possess many degrees of freedom (DoFs) beyond what is required to achieve desired movements (1). The question of controlling the vast amount of DoFs and executing coordinated whole-body movements remains an ongoing topic of inquiry in movement science and related fields. The center of body mass (CoM) is a critical state variable in whole-body movements as it reflects the movements of all body parts. Locomotion is a quintessential example of whole-body motion, and researchers have examined various features of CoM motion during locomotion. Computational models focusing on CoM can replicate several features of locomotion (2–6), including the transition from walking to running (7) and the ground reaction force during walking and running (8). The dynamic stability of locomotion relies on the relationship between the center of pressure (CoP) and extrapolated CoM (XCoM), which is a weighted sum of the position and velocity of CoM (9, 10). In amputee subjects, XCoM is farther from CoP on the prosthetic side than on the non-prosthetic side in the mediolateral direction (4), indicating that they prioritize dynamic stability on the prosthetic side rather than the non-prosthetic side. Thus, CoM is crucial in revealing fundamental features of whole-body motions, particularly during locomotion.

Achieving stable locomotion requires coordinated body motions in both the sagittal (including the anteroposterior and superoinferior directions) and frontal planes (including the mediolateral and superoinferior directions). However, computational models have shown that instability is greater in the frontal plane than in the sagittal plane (4–6), supported by experiments involving perturbations (11, 12). Additionally, elderly subjects with balance disorders and Parkinson's disease exhibit atypical CoM trajectories in the mediolateral direction (13, 14). Therefore, CoM motions in the mediolateral direction are critical in achieving stable locomotion. This study focuses on CoM trajectories in the frontal plane.

CoM trajectories in the frontal plane often exhibit figure-of-eight shapes (15–18). The figure-of-eight shapes can be decomposed into one frequency in the interval of one stride for the mediolateral motions (i.e., the first harmonic in the mediolateral direction) and two-frequency in the interval of one stride for the superoinferior motions [i.e., the second harmonic in the mediolateral direction, (19)]. Previous studies have shown speed-dependent changes in the figure-of-eight shape for symmetric CoM trajectories (15–18). Specifically, the width of the CoM trajectories decreases while the height increases as walking speed increases.

However, it is generally incorrect to assume that CoM trajectories are symmetric. Asymmetric CoM trajectories have been observed in amputee subjects (20). Patients with knee osteoarthritis demonstrate different accelerations of CoM movements in gait compared to healthy controls, indicating asymmetric CoM positions (21). As speed-dependent modulations of CoM trajectories were evaluated in symmetric CoM trajectories, it remains unclear how gait speed modulates asymmetric CoM trajectories.

In this study, our primary objective is to quantify the speed-dependent modulations of asymmetric CoM trajectories in individuals with above-knee amputations. While prior research has investigated dynamic stability, which is relevant to CoM position and velocity, as well as the speed-dependent modulations of dynamic stability in above-knee amputee subjects (4, 22), the specific modulations of temporally-varying CoM trajectories in this particular group of subjects have remained largely unexplored. Notably, Hood et al. (23) have graciously made their extensive gait data for above-knee amputee subjects available to the scientific community. Leveraging this valuable open dataset, we aim to elucidate the speed-dependent modulations of asymmetric CoM trajectories in individuals with above-knee amputations. We employ Fourier series expansion, enabling the assessment of periodic curves, including asymmetrical CoM trajectories, and facilitating the analysis of the functional impact of speed-dependent modulations [(20, 24), Supplementary Figure S1].

Firstly, we will confirm the asymmetry of CoM trajectories, which may arise from asymmetric stance duration between prosthetic and non-prosthetic legs (4). Secondly, we will use Fourier series expansion to evaluate the speed-dependent modulations of asymmetric CoM trajectories. Thirdly, we will examine the relationship between dynamic stability and walking speed based on the XCoM to confirm the functional role of the speed-dependent modulations. Previous research has reported that walking at faster speeds improves dynamic stability (22). Suppose speed-dependent modulations of CoM trajectories play a role in improving dynamic stability. In that case, we can expect the speed-dependent modulations of dynamic stability to be symmetric between the prosthetic and non-prosthetic sides. By validating this assumption, we will examine the functional role of changes in CoM trajectories depending on walking speed, which may guide amputee subjects to walk with more significant dynamic stability.

## Materials and methods

### Details of open data

In a previous study, gait data from above-knee amputee subjects walking on a treadmill at various speeds were made publicly available, and all relevant details are provided in (23). For the current study, we analyzed data from the eight amputee subjects who met the criteria of not grasping supportive bars beside the treadmill in all gait cycles and walking at five velocities (i.e., 0.6 m/s, 0.8 m/s, 1.0 m/s, 1.2 m/s, and 1.4 m/s). Although nine subjects met these criteria, motion capture data were missing in one subject, resulting in that we finally analyzed eight amputee subjects {age range: 23–61 [mean = 38.1, standard deviation = 12.6]; six males, two females; all at full community ambulator [K3 level of ambulation, as defined in the Medicare Functional Classification Level (MFCL) system, (25)] who can walk faster than 0.8 m/s (23) and with a prosthetic on the right side in three subjects}. Seven participants in the study employed microprocessor-controlled knee systems: three utilized the Plie FI, two used the C-Leg (manufactured by Ottobock, Duderstadt, Germany), and two opted for the Rheo Knee (manufactured by Össur, Reykjavik, Iceland). Additionally, one participant utilized a prosthetic knee joint equipped with hydraulic control for both the stance and swing phases of walking, identified as the 3R80 (manufactured by Ottobock, Duderstadt, Germany).

### Estimation of CoM position

The current study estimated the position of CoM based on the relative weight of each body part (26). Calculation of the mass of prosthetic legs and foots followed a previous study (27): The mass of the socket was set to 0.8 kg and the prosthetic shank and connectors to 0.3 kg. Based on manufacturer notices, the current study set the weight of prosthetic knees and feet. Because it was unclear whether the mass of the prosthetic knee included the mass of the socket, we added 0.2 kg to all the mass of prosthetic knees based on a description of 0.2 kg difference between empty weight and net weight in a manufacture notice of Rheo Knee. To calculate the mass of the prosthetic foot, we added 0.236 kg when there was no description of the mass of the footshell. 0.236 kg is an average weight across the clarified manufacture notes. Our results were insensitive to these weight assumptions, as shown in Supplementary Figures S3–S6. Note that the weight of shoes was not provided in the open data (23), but it does not affect our main results (Supplementary Figures S7–S10). Throughout this paper, we set the weight of shoes to 0.

To estimate CoM in amputee subjects, we had to adjust the method used in (26), which is only valid for non-amputee subjects. We defined the total weight of the prosthetic knee and foot as “m.” The weight of the amputee subject, “w,” is equal to (1−0.161)M+m, where 0.161M is the relative mass of the total non-prosthetic leg and *M* is the supposed weight of amputee subjects when they are hypothetically non-amputees. Thus, we can estimate *M* as M=(w−m)/0.839 for amputee subjects and calculate CoM based on the weight of each body part relative to *M*. For instance, the relative weight of the thigh is 0.1M in non-amputee subjects, while it is 0.1(w−m)/0.839 in amputee subjects. We set the CoM of the prosthetic leg to 25% below the top of the shank (23, 28). The CoM of the prosthetic foot and thigh was set to be the same as each non-prosthetic part.

After estimating the CoM position, we segmented it into individual strides, from right foot contact to the next right foot contact. In this study, a single gait cycle is defined as the duration from right foot contact to the subsequent right foot contact (i.e., one stride). Then, to remove the fluctuation effect in the stance position, we subtracted the mean CoM position from the CoM position in each stride. Next, we normalized the number of time frames in each stride to 200 frames. Finally, to eliminate any outliers, we excluded gait cycles identified by “rmoutliers” in MATLAB2022a (The MathWorks Inc., Natick, Massachusetts) from the subsequent analysis.

### Fourier series expansion of CoM trajectories

The Fourier series expansion applies to time series data whose initial value equals to terminal value. To apply the Fourier series expansion, we averaged CoM trajectories across all gait cycles in each speed and subject (averaged number of gait cycles across subjects was 69.3, 71.8, 73.9, 78.5, and 78.5, in the gait at 0.6 m/s, 0.8 m/s, 1.0 m/s, 1.2 m/s, and 1/4 m/s, respectively) and made initial and terminal values the same using the previous method (24). In the mediolateral direction, CoM trajectories could be accurately described using only the first harmonic ($R^{2}\;>\;0.99$). This observation suggests that CoM trajectories began at the initial position during right foot contact, moved rightward, then leftward, and finally returned to a position close to the starting point upon the subsequent right foot contact. Detailed examples of Fourier series expansion illustrating this pattern are provided in Supplementary Figure S1.

Conversely, in the suprainferior direction, achieving a comparable fitting performance as observed in the mediolateral direction necessitated the inclusion of the first to the fourth harmonics. Specifically, the second harmonic indicated that CoM trajectories exhibited upward and downward movements within a single step, transitioning from right to left heel contact, and again during the step from left to right heel contact, as illustrated in Supplementary Figure S1. Notably, the presence of the second and fourth harmonics suggested symmetric CoM trajectories between the right and left legs, while the first and third harmonics indicated asymmetric CoM trajectories (see Supplementary Figure S1).

We thus analyzed the first harmonic in the mediolateral direction and the first to fourth harmonics in the suprainferior direction as follows:

(1) $$\mathrm{CO}M_{y,t}\;=\;\overset{4}{\underset{n=1}{\sum }}\left(a_{y,nc}\cos\left(2n\pi \frac{t}{T}\right)\;+\;a_{y,ns}\sin\left(2n\pi \frac{t}{T}\right)\right)\;=\;\overset{4}{\underset{n=1}{\sum }}r_{y,n}\cos\left(2n\pi \frac{t}{T}-\delta_{y,n}\right),$$

and

(2) $$\mathrm{CO}M_{z,t}\;=\;a_{z,1c}\cos\left(2\pi \frac{t}{T}\right)\;+\;a_{z,1s}\sin\left(2\pi \frac{t}{T}\right)\;=\;r_{z,1}\cos\left(2\pi \frac{t}{T}-\delta_{z,1}\right),$$

where $\mathrm{CO}M_{y,t}$ is the positions of CoM in the superoinferior direction at normalized time t(t=0,…,200), $a_{y,nc}\;=\;\frac{2}{T}\int_{0}^{T}\mathrm{CO}M_{y,t}\cos\left(2n\pi \frac{t}{T}\right)dt\;$ is the coefficient for the cosine function of nth harmonic, $a_{y,ns}\;=\;\frac{2}{T}\int_{0}^{T}\mathrm{CO}M_{y,t}\sin\left(2n\pi \frac{t}{T}\right)dt\;$ is the coefficient for the sinusoidal function of nth harmonic, $r_{y,n}\;=\;\sqrt{a_{y,nc}^{2}\;+\;a_{y,ns}^{2}\;}\;$ is the amplitude of nth harmonic, $\delta_{y,n}\;=\;\cos^{-1}\left(\frac{a_{y,nc}}{r_{y,n}}\right)\;$ is the phase delay in *n* th harmonic, and T=200 is the length of normalized time frames in each stride. In $\mathrm{CO}M_{z,t}$, the CoM positions in the mediolateral direction, the definitions of all variables are the same as those in $\mathrm{CO}M_{y,t}$. The current study focuses on how walking speed modulates *r* and δ.

### Calculation of XCoM, CoP, and step width

We calculated XCoM in each stride without normalizing time frames as

(3) $$\mathrm{XCO}M_{z,t}\;=\;\;\mathrm{CO}M_{z,t}\;+\;\frac{1}{w_{0}}\frac{d}{dt}\mathrm{CO}M_{z,t},$$

where $w_{0}\;=\;\;\sqrt{\frac{g}{l}}$, $g\;=\;9.8\;(m/s^{2})$, and *l* is the leg length. The difference between XCoM and CoP in the mediolateral direction is a determinant of dynamic stability (10), and a smaller difference indicates greater dynamic instability. CoP was estimated based on the ankle joint's position in the contacted foot, and the margin of stability (MoS) was calculated as the minimum distance between XCoM and CoP. On the prosthetic side, we approximated CoP as the center of ankle position in the prosthetic leg, as denoted in (23). Step width was calculated as the difference between the CoP on the prosthetic and non-prosthetic side within one stride. We ensured that XCoM was within the area between left and right CoPs at foot contact. Finally, we calculated the average MoS across gait cycles at each walking velocity.

Under the framework of Fourier series expansion and normalized time across all the strides (Equation 2), XCoM can be approximated as

(4) $$\mathrm{XCO}M_{z,t}\;=\;r_{z,1}\left(\cos\left(2\pi \frac{t}{T}-\delta_{z,1}\right)-\frac{1}{w_{0}}\sin\left(2\pi \frac{t}{T}-\delta_{z,1}\right)\right)\;,$$

indicating that MoS depends on $r_{z,1}$ and step width.

### Statistical analysis

We used a 2-way repeated measures ANOVA to analyze foot contact duration and MoS, with walking velocity and prosthetic/non-prosthetic side as independent variables. We used a 1-way repeated measures ANOVA with walking velocity as the independent variable for amplitudes rs and phases δs (Equations 1, 2). To account for individual differences due to the variability of prosthetic devices across subjects, we included subject ID as a random effect in the ANOVA.

## Results

**Figure 1:**
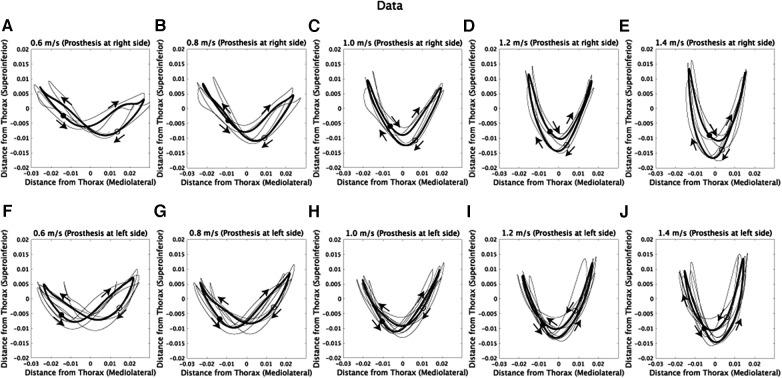
Com trajectories of amputee subjects at five walking speeds. The *x*-axis represents the horizontal position, where positive values denote the right side of the subjects, and negative values denote the left side. (**A–E**) CoM trajectories of subjects with a prosthetic limb on the right side while walking at 0.6, 0.8, 1.0, 1.2, and 1.4 m/s. Each black line represents the trajectory of an individual subject, and the thick black line indicates the average trajectory across subjects. Filled circles represent the CoM position at the initial time frame (1st), while hollow circles represent the position at the half of one stride (100th). Additionally, arrows are used to illustrate the direction of CoM velocities within the frontal plane. (**F–J**) CoM trajectories of subjects with a prosthetic limb on the left side.

The walking patterns of amputee subjects exhibit asymmetric CoM trajectories, as shown in Figure 1. Specifically, in amputee subjects with a prosthetic on the right side, the CoM trajectories lean towards the lower-right direction (Figures 1A–D). The opposite is observed for amputee subjects with a prosthetic on the left side (Figures 1E–H).

**Figure 2:**
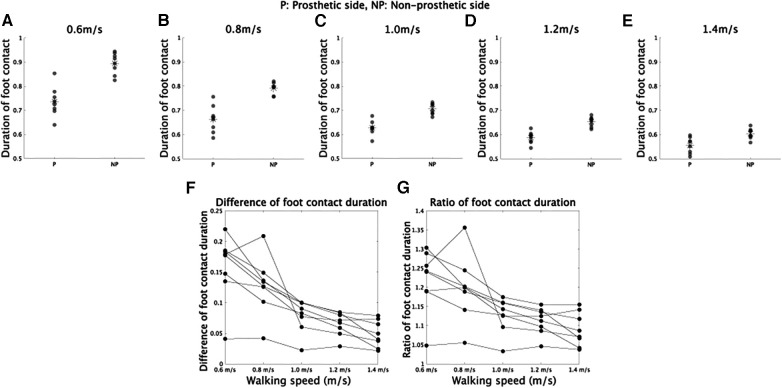
Foot contact duration in prosthetic and non-prosthetic sides. (**A–E**) Foot contact duration in walking at 0.6, 0.8, 1.0, 1.2, and 1.4 m/s, respectively. (**F**) Difference of foot contact duration between prosthetic and non-prosthetic sides in each walking speed. (**G**) Ratio of foot contact duration in each walking speed.

The asymmetry observed in CoM trajectories might be due to the asymmetric durations of foot contacts between the prosthetic and non-prosthetic sides, as depicted in Figure 2 and described in previous studies (4, 29, 30). We conducted repeated measures ANOVA on foot contact durations (Figures 2A–D), considering both walking velocity and the prosthetic or non-prosthetic side as independent variables. We found significant main effects of velocity [*F*(4,28) = 317.5, $p\;=\;\;6.9\;\times \;10^{-23}$]*,* the prosthetic or non-prosthetic side [*F*(1,7) = 91.2, $p\;=\;\;2.9\;\times \;10^{-5}$], and an interaction between the two variables [*F*(4,28) = 25.8, $p\;=\;\;4.8\;\times \;10^{-9}$]. The difference in foot contact duration between the prosthetic and non-prosthetic sides was significantly different from 0 at all speeds (0.6 m/s: *p* = 0.00028, 0.8 m/s: *p* = 0.00044, 1.0 m/s: *p* = 0.00027, 1.2 m/s: *p* = 0.00011, and 1.4 m/s: *p* = 0.0015, all *p*-values were corrected), indicating that the duration of foot contact was shorter on the prosthetic side compared to the non-prosthetic side. Furthermore, there was a significant main effect of velocity on the difference [Figure 2E, *F*(4,28) = 25.8, $p\;=\;\;4.8\;\times \;10^{-9}$] and the ratio of durations [Figure 2F, *F*(4,28) = 14.2, $p\;=\;\;1.9\;\times \;10^{-6}$]. Our findings are consistent with those of previous studies (30), which suggest that walking speed modulates the asymmetry of foot contact time [cf. the discussion in (29)].

**Figure 3:**
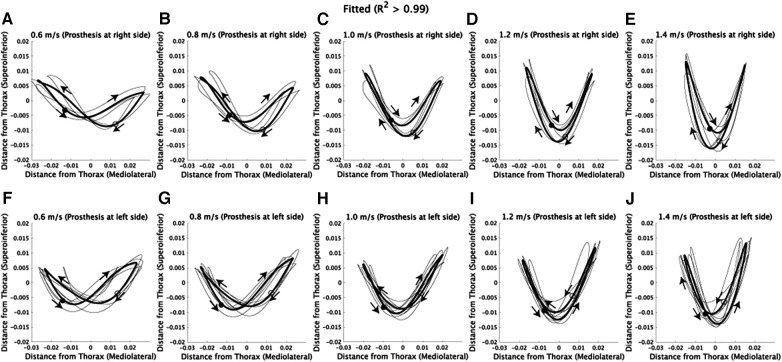
Fitted CoM trajectories via Fourier series expansion. The x-axis represents the horizontal position, where positive values denote the right side of the subjects, and negative values denote the left side. (**A–E**) CoM trajectories fitted to the corresponding original CoM trajectories shown in Figure 1A–E. Each thin black line represents the fitted trajectory of an individual subject, and the solid lines indicate the average of fitted trajectories. Filled circles represent the CoM position at the initial time frame (1st), while hollow circles represent the position at the half of one stride (100th). Additionally, arrows are used to illustrate the direction of CoM velocities within the frontal plane. (**F–J**) CoM trajectories fitted to the corresponding original CoM trajectories shown in Figure 1F–J.

We employed Fourier series expansion to examine how walking speed affects the asymmetry of CoM trajectories [Supplementary Figure S1 (20, 24)]. The mediolateral CoM trajectories can be well represented by a the first harmonic ($R^{2}\;>\;0.99$, Supplementary Figure S2). Therefore, we selected the number of components in the Fourier series expansion based on the criterion of $R^{2}\;>\;0.99$. For the superoinferior direction, the first or third harmonic can be used to model the asymmetric CoM trajectories (Supplementary Figure S1), and combining from one to four frequencies allowed us to model the asymmetric CoM trajectories with an $R^{2}\;>\;0.99$ (Supplementary Figure S2). Each panel in Figure 3A–J displays the CoM trajectories fitted to the corresponding original CoM trajectories shown in Figure 1A–J. As a result, the fitted CoM trajectories exhibit similar patterns and trends to the original CoM trajectories.

**Figure 4:**
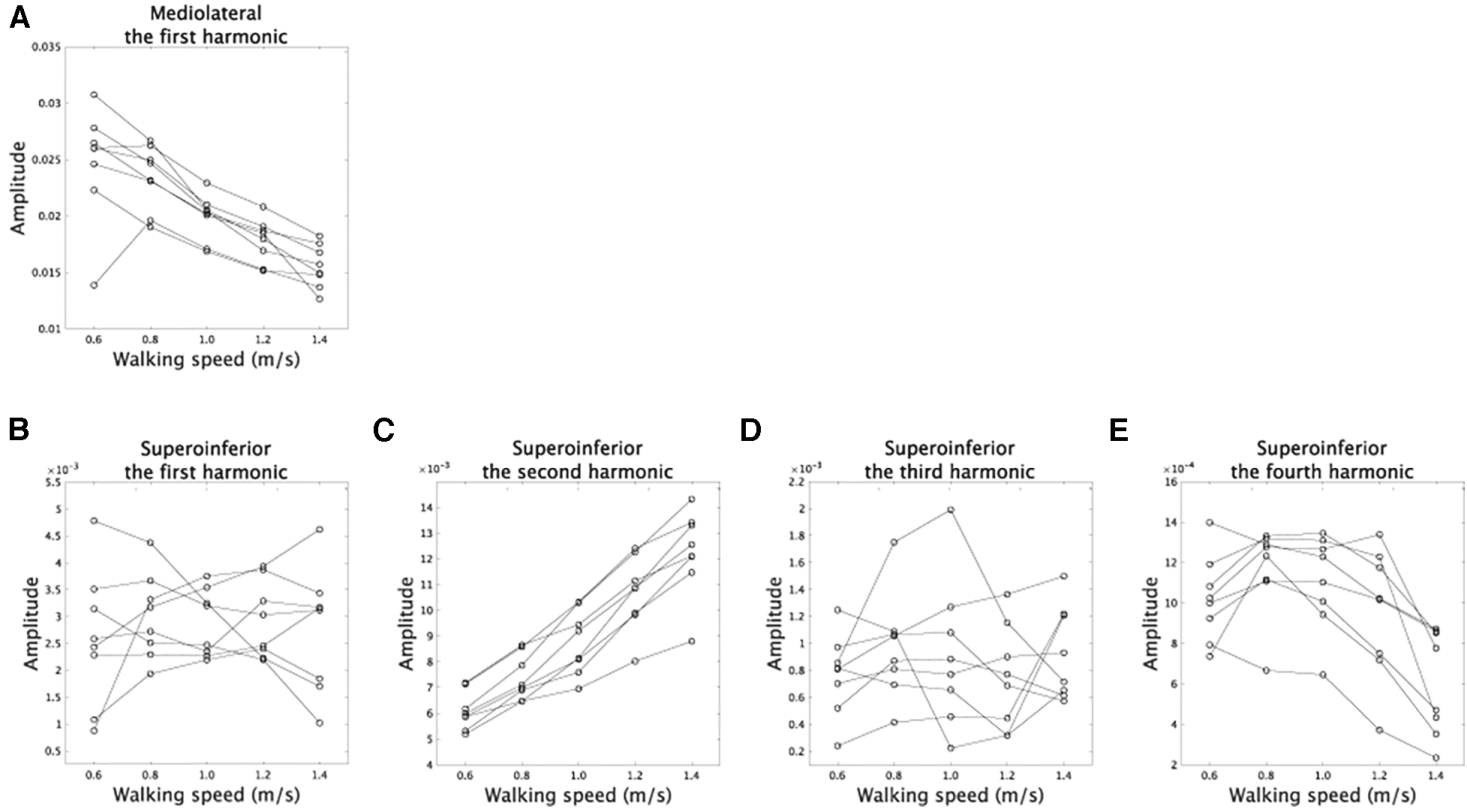
Speed-dependent modulations of amplitudes. (**A**) The modulations of the amplitudes in the first harmonic in the mediolateral direction. (**B–E**) The speed-dependent modulations of the amplitudes in the first, second, third, and fourth harmonics in the superoinferior direction.

The amplitudes in Fourier series expansion (r in Equations 1, 2) quantified how walking speed modulates asymmetric CoM trajectories. In the mediolateral direction, the amplitude of the the first harmonic showed a speed-dependent modulation or a main effect of velocity (Figure 4A, *F*(4,28) = 27.9, $p=\;2.1\times 10^{-9}$ [corrected]). In the superoinferior direction, the amplitudes of the second and fourth harmonics showed speed-dependent modulation [Figure 4B–E *F*(4,28) = 97.0, $p\;=\;\;2.0\;\times \;10^{-15}$ [corrected] for second harmonic, *F*(4,28) = 21.0, $p\;=\;\;1.8\;\times \;10^{-7}$ [corrected] for fourth harmonic, and *F*(4,28) < 0.67, *p* > 0.62 [uncorrected] for other components]. These results are similar to those found in non-amputee subjects (18): the amplitude in the first harmonic in the mediolateral direction decreased as walking speed increased, and the amplitudes in the second harmonic in the superoinferior direction increased in proportion to walking speed. In amputee subjects, speed-dependent modulations exhibit statistical significance in the fourth harmonic. However, it's worth noting that this modulation does not have an impact on CoM trajectories, as shown in the Discussion section. These results indicate that CoM trajectories become narrower in the mediolateral direction and broader in the superoinferior direction at faster walking speeds in both amputee and non-amputee subjects.

**Figure 5:**
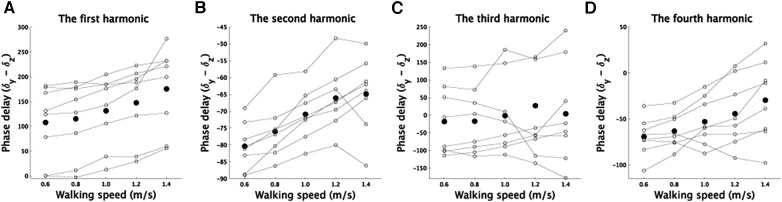
Modulations in phases (degrees) related to the superoinferior direction for each component as a function of walking speed. (**A–D**) The speed-dependent modulations of the phases in the first, second, third, and fourth harmonics in the superoinferior direction. The vertical axis shows the difference between the phase of each component and the phase of the first harmonic related to the mediolateral direction. The phase difference is crucial in determining the shape of the fitted curve (18), and thus we included it in the figure. The plot illustrates the speed-dependent modulations in phase for each component. Solid lines denote the phase modulations in each subject, and black dots show the average across the subjects.

The phases in Fourier series expansion (Equations 1, 2) determine the shape of CoM trajectories at each walking speed (Supplementary Figure S1). In this study, we calculated the difference between $\delta_{y}$ and $\delta_{z}$ in Equations 1, 2 because the phase difference affects CoM trajectory shape (18). In the mediolateral direction, $\delta_{z}$ represents the phase in the the first harmonic, while in the superoinferior direction, $\delta_{y}$ represents the phase in the first, second, third, or fourth harmonic. Although there was no significant speed-dependent modulation in the third harmonic [Figure 5C, *F*(4,28) = 0.36, *p* = 0.83], there were significant speed-dependent modulations in other components [Figure 5A, B, D, *F*(4,28) = 17.1, $p\;=\;\;1.4\;\times \;10^{-6}$ [corrected] for the first harmonic, *F*(4,28) = 20.6, $p\;=\;\;2.1\;\times \;10^{-7}$ [corrected] for the second harmonic, and *F*(3,21) = 6.3, *p* = 0.0039 [corrected] for the fourth harmonic].

We then investigated the functional significance of speed-dependent modulations of CoM trajectories. In the mediolateral direction, dynamic stability is determined by the difference between CoP and XCoM (Equation 3), which is known as MoS (10). Within our framework, XCoM is dependent on the amplitude of the first harmonic in the mediolateral direction (Equation 4). Assuming a constant step width across walking speeds, we can expect that speed-dependent modulations of CoM trajectories serve to improve dynamic stability while reducing the width of CoM trajectories and increasing MoS.

**Figure 6:**
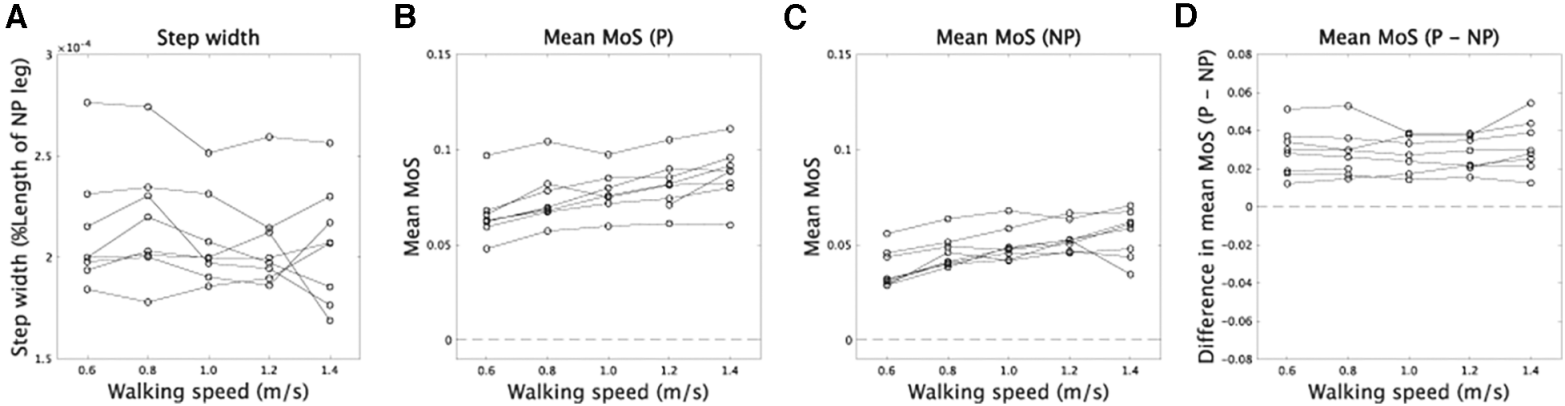
Step width and MoS. (**A**) The relation between walking speed and step width. (**B,C**) The relation between walking speed and MoS in prosthetic (P) and non-prosthetic (NP) sides, respectively. (**D**) Asymmetry of MoS between prosthetic and non-prosthetic sides.

Our analysis found no significant main effect of velocity on step width, suggesting that step width remained relatively constant across different walking speeds [Figure 6A, *F*(4,28) = 1.16, *p* = 0.35]. To determine if faster walking speeds improved dynamic stability, we conducted repeated measures ANOVA on MoS. As anticipated, we observed a significant main effect of velocity on MoS [*F*(4,24) = 30.3, $p\;=\;\;4.6\;\times \;10^{-9}$], as well as a significant main effect of whether the side was prosthetic or non-prosthetic [*F*(1,6) = 51.5, $p\;=\;\;3.7\;\times \;10^{-4}$]. However, there was no interaction between these two independent variables [*F*(4,24) = 0.78, *p* = 0.55]. A significant main effect of velocity would indicate that modulations of CoM width related to walking speed contribute to greater dynamic stability at faster walking speeds. Our results also showed that dynamic stability was higher on the prosthetic side compared to the non-prosthetic side (Figure 6), consistent with previous findings (4) [cf (22)].

Suppose the speed-dependent modulations of CoM trajectories play a role in increasing dynamic stability at a faster walking speed. In that case, we can expect the asymmetry of dynamic stability to be invariant across walking speed (Equation 4). In our framework, XCoM depended on the amplitude of the first harmonic that is symmetric between prosthetic and non-prosthetic sides. According to this assumption, there was no significant main effect of velocity in the difference of MoS between prosthetic and non-prosthetic sides [*F*(4,24) = 0.78, *p* = 0.55], indicating the asymmetry of dynamic stability did not depend on velocity.

## Discussion

We used Fourier series expansion (Figures 1, 3, 4) to demonstrate speed-dependent modulations of asymmetric CoM trajectories in above-knee amputee subjects. Asymmetric foot contact durations would cause asymmetric CoM trajectories (Figures 1, 2). Interestingly, the speed-dependent modulations in amputee subjects were similar to those in non-amputee subjects [Figure 4, (18)], where the amplitude of the first harmonic in the mediolateral direction decreased, and the amplitude of the second harmonic in the superoinferior direction increased as walking speed increased (Figure 4). In other words, trajectories became narrower in the mediolateral direction and broader in the superoinferior direction at faster walking speeds (15–18), which was consistent across amputee and non-amputee subjects. Additionally, the speed-dependent modulations in the first harmonic in the mediolateral direction were related to greater dynamic stability at faster walking speeds, as evidenced by larger MoS and invariant asymmetry of dynamic stability (Figure 6). Maintaining higher stability on the prosthetic side may lead to a greater reliance on the non-prosthetic side. While amputee subjects can rapidly and actively adapt their movements to improve stability on the non-prosthetic side, achieving flexible stability control on the prosthetic side can be challenging, primarily due to factors such as the loss of active knee extension in the prosthetic limb. One potential approach to achieving a stable gait is to enhance dynamic stability on the prosthetic side and employ adaptable strategies to compensate for disturbances on the non-prosthetic side.

The speed-dependent modulations in the second harmonic in the superoinferior direction indicated that larger gravitational potential energy in the vertical direction was associated with faster walking speeds. The kinetic energy for progression, $K_{p}$, showed a mirror image against the sum of gravitational potential energy and kinetic energy in the vertical direction, $T_{v}$ (31–33), suggesting that larger gravitational potential energy was linked to faster progression. Our results indicated that this mirror image between $T_{v}$ and $K_{p}$ was invariant in amputee subjects on both prosthetic and non-prosthetic sides. One possible action policy was to increase gravitational potential energy only on the non-prosthetic side by modulating the first harmonic in the superoinferior direction (Supplementary Figure S1). However, the amputee subjects modulated their walking speed to obtain gravitational potential energy in both prosthetic and non-prosthetic sides (Figure 4).

**Figure 7:**
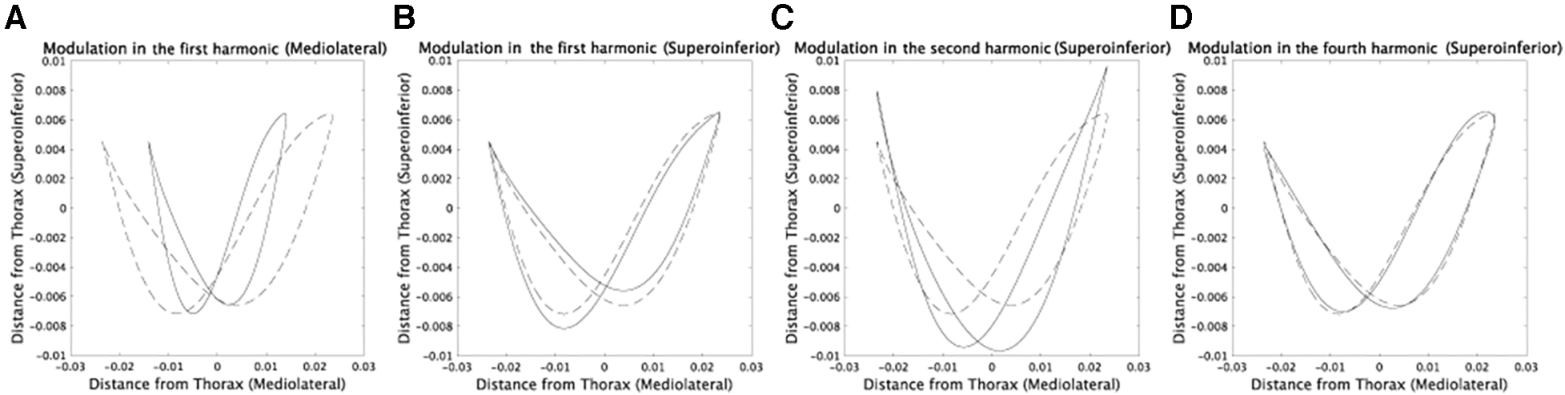
Detailed investigation of speed-dependent modulations in each component via comparing the CoM trajectories at 0.6 m/s and 1.2 m/s. Dotted lines indicate averaged CoM trajectories in subjects whose prosthetic sides are left at 0.6 m/s. (**A**) Solid lines denote the CoM trajectories with speed-dependent modulations in amplitudes and phases only in the first harmonic in the mediolateral direction. We calculated the modulations by averaging the values at 1.2 m/s in Figures 4, 5. (**B**) The modulations in phases only in the first harmonic component in the superoinferior direction. (**C**) The modulations in amplitudes and phases only in the second harmonic in the superoinferior direction. (**D**) The modulations in phases only in the fourth harmonic in the superoinferior direction.

The results shown in Figure 7 allowed for a detailed examination of the functional roles of the speed-dependent modulations. In the mediolateral direction, the speed-dependent modulation in amplitude (Figure 4A) led to narrower CoM trajectories (Figure 7A). In the superoinferior direction, the modulation in the phase of the first harmonic (Supplementary Figure S1A) emphasized the asymmetry of CoM trajectories while lowering and raising the CoM position in prosthetic and non-prosthetic sides, respectively (Figure 7B). The observed phase modulation led to an increased energy requirement for raising the CoM position on the prosthetic side. This suggests a scattering of $T_{v}$ during the raising phase and a decrease in $K_{p}$. In contrast, on the non-prosthetic side, an efficient transformation of $T_{v}$ into $K_{p}$ was feasible due to the lower energy requirement for raising the CoM position. Overall, the first harmonic's phase modulation in the superoinferior direction may indicate a faster progression speed on the non-prosthetic side. The speed-dependent modulations of the second harmonic modulated the trajectories to be symmetric (Figure 7C). The modulation of the fourth harmonic induced a subtle change (Figure 7D), and this modulation was found to be sensitive to shoe weight (Supplementary Figures S7, S9). In summary, amputee subjects increased $K_{p}$ while increasing gravitational potential energy more on the prosthetic than on the non-prosthetic side (Figure 7B and C).

We have provided a summary of the consistency and inconsistency of our results with previous findings, as there were conflicting reports in the literature. Our study found that foot contact duration is shorter on the prosthetic side than on the non-prosthetic side, which is consistent with (29, 30) (Figure 2). However, our results support that the asymmetry of foot contact time decreases as walking speed increases, contrary to the constant asymmetry reported in (29). In terms of MoS, our analysis found that MoS was smaller on the non-prosthetic side than on the prosthetic side, which is consistent with (4) (Figure 6). However, the opposite tendency was reported in (22). Our study found that MoS increases as walking speed increases, which is consistent with (22) (Figures 6B,C). Notably, we found that the asymmetry of MoS is invariant across walking speed (Figure 6D).

One limitation of this study is the absence of consideration regarding the type of prosthetic knee, such as a comparison between microprocessor-controlled (MPC) knees and other prosthetic knee types. Since seven out of eight participants utilized MPC knees in our analysis, a significant portion of our results is based on MPC knees. In the comparison between participants with MPC knees and one participant with a prosthetic knee joint utilizing hydraulic control, we observed differences in the amplitude of Fourier components (Supplementary Figure S11) and MoS (Supplementary Figure S12). However, this comparison lacks reliability due to the analysis being based on a single subject with a non-MPC knee. Future research is needed to investigate the impact of prosthetic knee type on CoM trajectories and dynamic stability.

In this study, our primary focus was on gait within the speed range of 0.6–1.4 m/s. However, it is essential to explore whether our findings remain consistent at higher gait speeds, as suggested by Latt et al. (34), to ensure the generalizability of our results. Furthermore, we assessed gait stability, specifically dynamic stability, with a particular emphasis on MoS. It is worth noting that further research is needed to provide a comprehensive interpretation of dynamic stability within the scope of our study, as well as its relationship with other aspects of stability, as proposed by Dingwell and Marin (35).

To better understand our results from kinematic and kinetic perspectives, we suggest discussing the relationship between CoM trajectories and the kinematics and kinetics of each body part. The analyzed dataset (23) includes motion data measured from several body parts, making it possible to estimate how each joint angle affects CoM trajectories, for example, using data-driven methods (36, 37). These methods can also estimate how prosthetic knee and ankle joint angles affect CoM trajectories. Additionally, investigating the coordinated group of joint angles or muscles is an interesting topic. Although the functional roles of these groups are typically examined in non-amputee subjects (38–41), future studies could explore the roles of joint angle and muscle groups in amputee subjects.

## Data Availability

The original contributions presented in the study are included in the article/**Supplementary Material**, further inquiries can be directed to the corresponding author.
