# Supplementary material for: Speed-dependent modulations of asymmetric center of body mass trajectory in the gait of above-knee amputee subjects
Source: Front Sports Act Living. 2024 Jan 4;5:1304141. doi: 10.3389/fspor.2023.1304141 (PMC10794564; doi:10.3389/fspor.2023.1304141)
Supplement: Supplementary file 1 [file Datasheet1.pdf]

Supplementary materials for Speed-dependent modulations of asymmetric CoM trajectory in gait of above-knee amputee subjects

Ken Takiyama*[a], Hikaru Yokoyama[b]

[a] Tokyo University of Agriculture and Technology, Department of Electrical Engineering and Computer Science, Nakacho, Koganei, Tokyo, Japan

[b] Tokyo University of Agriculture and Technology, Division of Advanced Health Science, Nakacho, Koganei, Tokyo, Japan

Corresponding author: Ken Takiyama, e-mail address: ken-taki@cc.tuat.ac.jp

An example of Fourier series expansion

The Fourier series expansion provides a means to model asymmetric curves by adjusting the amplitudes and phases of each cyclic component (eqs. 1 and 2). In our study, we were able to fit the CoM trajectory data with high accuracy, achieving R^2 values greater than 0.99 (Fig. S2) by utilizing up to four frequencies within one stride interval.

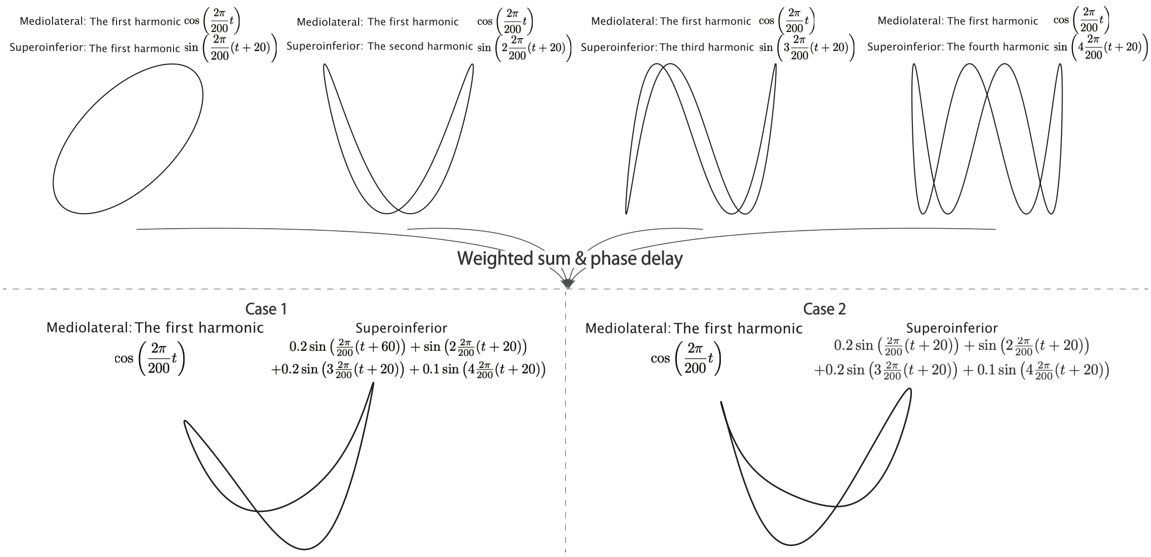

Fig. S1: Concept of Fourier series expansion. While fixing amplitude and phase in the first harmonic of the mediolateral direction, we change other parameters.

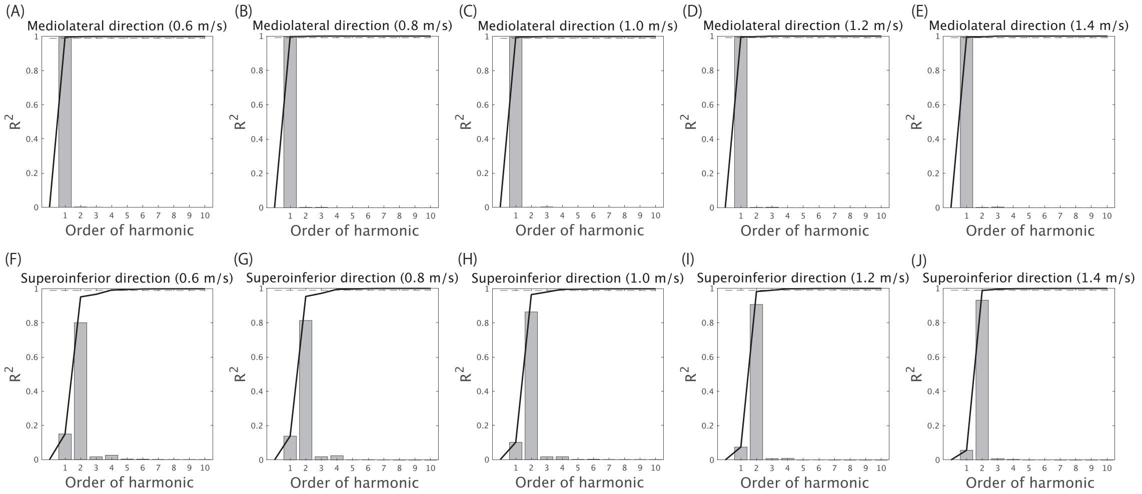

Fig. S2: fitting performance of each component. Bar graphs indicate R^2 by using only the corresponding component. Solid lines indicate R^2 by using up to the denoted order of harmonic (e.g., the solid line at the point of the second harmonic means R^2 by using the first and second harmonics).

The influence of mass of socket

The following results were when we did not add 0.2kg as a socket mass to participants whose prosthetic knee is not Rheo Os. We set the shoe weight to be 0 because the shoe weight does not affect our main results (Figs. S7-10).

There was a significant main effect of velocity in the first harmonic in the mediolateral direction (F(4,28) = 27.9, $p = 2.1 \times 10^{-9}$), the second harmonic in the superoinferior direction (F(4,28) = 96.9, $p = 1.0 \times 10^{-15}$ [uncorrected], and the fourth harmonic in the superoinferior direction (F(4,28) = 20.9, $p = 4.6 \times 10^{-8}$). There was no main effect of velocity on the first harmonic in the superoinferior direction (F(4,28) = 0.29, p = 0.88 [uncorrected]) and the third harmonic in the superoinferior direction (F(4,28) =0.67, p = 0.62 [corrected]). The mass of the socket does not affect the speed-dependent modulations in the amplitudes of each component.

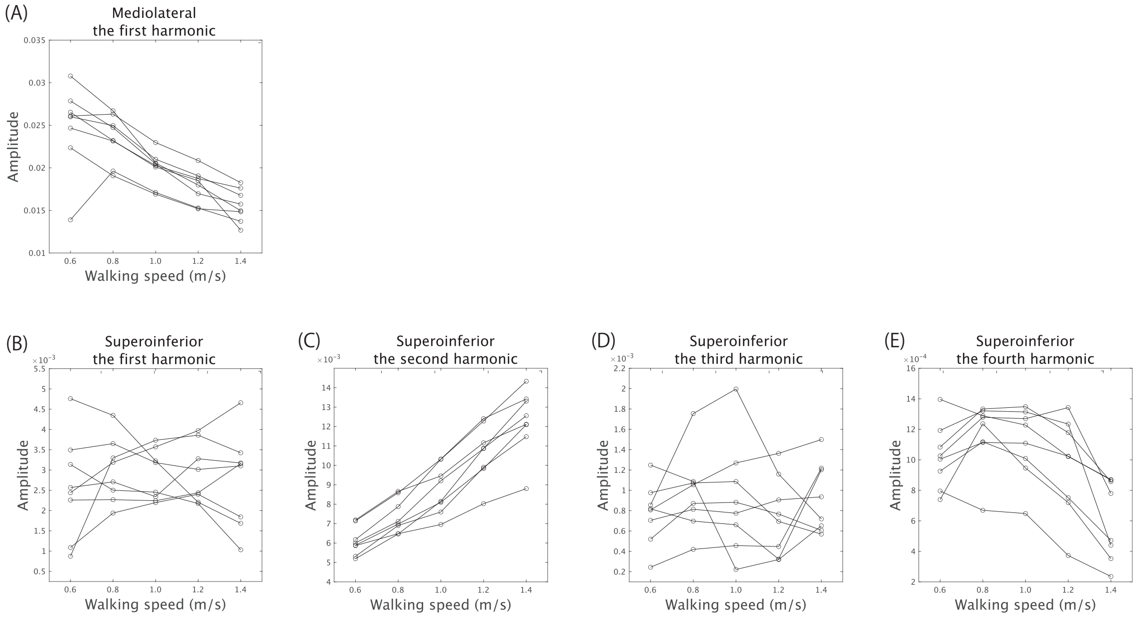

Fig. S3: Speed-dependent modulations of amplitudes in each component when we did not add the socket mass to participants whose prosthetic knee is not Rheo Os. (A): The modulations in the first harmonic in the mediolateral direction. (B-E): The speed-dependent modulations in the first, second, third, and fourth harmonics in the superoinferior direction.

In the analysis of MoS, there was a significant main effect of velocity (F(4,24) = 30.5, $p = 4.4 \times 10^{-9}$) and either prosthetic or non-prosthetic side (F(1,6) = 55.5, $p = 3.0 \times 10^{-4}$). There was no interaction between velocity and prosthetic/non-prosthetic side (F(4,24) = 0.78, p = 0.55). There was no main effect of velocity on the asymmetry of MoS (F(4,24) = 0.78, p = 0.55). The mass of the socket does not affect the speed-dependent modulations in MoS.

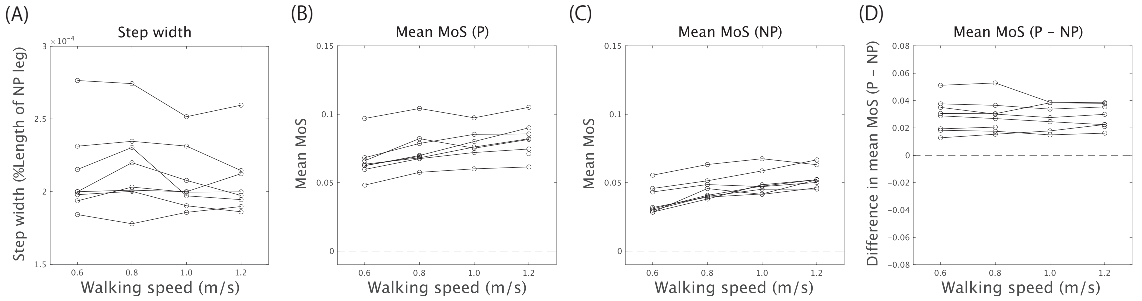

Fig. S4: Step width and MoS when we did not add the socket mass to participants whose prosthetic knee is not Rheo Os. (A): The relation between walking speed and step width. (B, C): The relation between walking speed and MoS in prosthetic (P) and non-prosthetic (NP) sides, respectively. (D): Asymmetry of MoS between prosthetic and non-prosthetic sides.

The influence of mass of footshell

The following results were the case when we did not add 0.236kg as a mass of footshell to participants when there was no description of the mass of footshell. We set the shoe weight to be 0 because shoe weight does not affect our main results (Figs. S7-10).

There was a significant main effect of velocity in the first harmonic in the mediolateral direction (F(4,28) = 28.0, $p = 2.0 \times 10^{-9}$), the second harmonic in the superoinferior direction (F(4,28) = 96.7, $p = 1.0 \times 10^{-15}$ [uncorrected]), and the fourth harmonic in the superoinferior direction (F(4,28) = 20.9, $p = 4.6 \times 10^{-8}$ [uncorrected]). There was no main effect of velocity on the first harmonic in the superoinferior direction (F(4,28) = 0.29, p = 0.88 [uncorrected]) and the third harmonic in the superoinferior direction (F(3,21) =0.66, p = 0.63 [uncorrected]). The mass of footshell does not affect the speed-dependent modulations in amplitudes of each component.

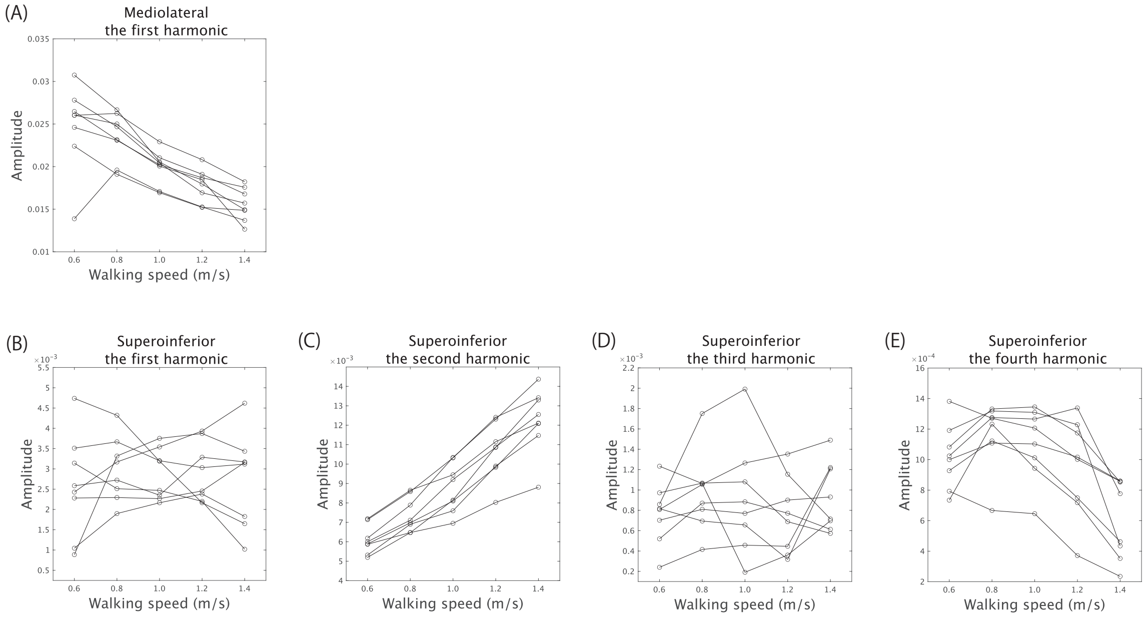

Fig. S5: Speed-dependent modulations of amplitudes in each component without additional mass of footshell when there is no description of the mass. (A): The modulations in the first harmonic in the mediolateral direction. (B-E): The speed-dependent modulations in first, second, third, and fourth harmonics in the superoinferior direction.

In the analysis of MoS, there was a significant main effect of velocity (F(4,24) = 30.3, $p = 4.6 \times 10^{-9}$) and either prosthetic or non-prosthetic side (F(1,6) = 50.0, $p = 4.0 \times 10^{-4}$). There was no interaction between velocity and prosthetic/non-prosthetic side (F(4,24) = 0.78, p = 0.54). There was no main effect of velocity on the asymmetry of MoS (Asymmetry; F(4,24) = 0.78, p = 0.55). The mass of footshell does not affect the speed-dependent modulations in MoS.

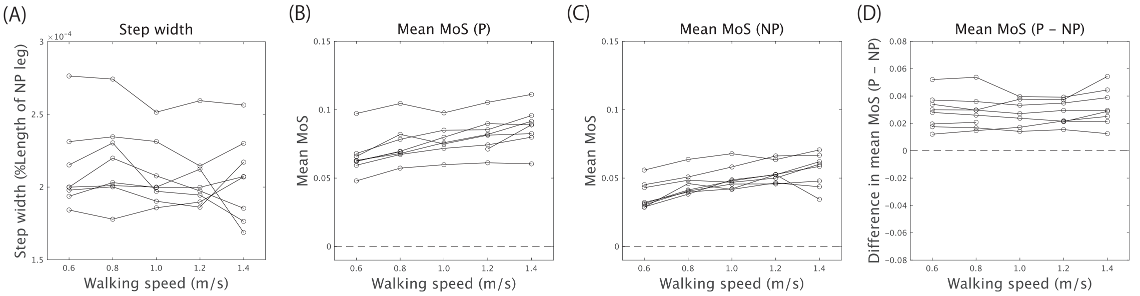

Fig. S6: Step width and MoS without additional mass of footshell when there is no description of the mass. (A): The relation between walking speed and step width. (B, C): The relation between walking speed and MoS in prosthetic (P) and non-prosthetic (NP) sides, respectively. (D): Asymmetry of MoS between prosthetic and non-prosthetic sides.

The influence of shoe weight on our results

We investigated the effect of shoe weight by adding either 0.3kg or 0.6kg to the weight of the foot. Since there was no information regarding shoe weight in the dataset [22], we assumed that shoe weight was uniform across all participants in the study.

The case when shoe weight is 0.3(kg)

There was a significant main effect of velocity in the first harmonic in the mediolateral direction (F(4,28) = 27.8, $p = 2.2 \times 10^{-9}$), the second harmonic in the superoinferior direction (F(4,28) = 96.2, $p = 1.0 \times 10^{-15}$ [uncorrected]), and the fourth harmonic in the superoinferior direction (F(4,28) = 19.6, $p = 8.7 \times 10^{-8}$ [uncorrected]). There was no main effect of velocity on the first harmonic in the superoinferior direction (F(4,28) = 0.32, p = 0.86 [uncorrected]) and the third harmonic in the superoinferior direction (F(4,28) =0.69, p = 0.60 [uncorrected]). The shoe weight does not affect the speed-dependent modulations in amplitudes of each component when the weight is 0.3.

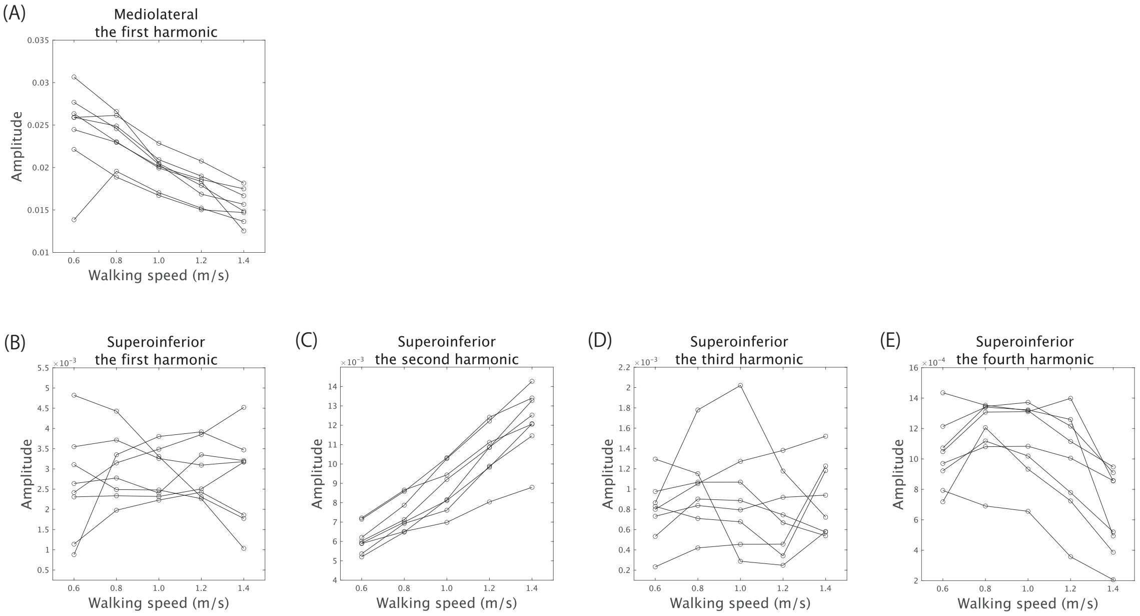

Fig. S7: Speed-dependent modulations of amplitudes in each component when shoe weight is 0.3(kg). (A): The modulations in the first harmonic in the mediolateral direction. (B-E): The speed-dependent modulations in the first, second, third, and fourth harmonics in the superoinferior direction.

In the analysis of MoS, there was a significant main effect of velocity (F(4,24) = 30.0, $p = 5.1 \times 10^{-9}$) and either prosthetic or non-prosthetic side (F(1,6) = 47.3, $p = 4.7 \times 10^{-4}$). There was no interaction between velocity and prosthetic/non-prosthetic side (F(4,24) = 0.79, p = 0.54). There was no main effect of velocity on the asymmetry of MoS (Asymmetry; F(4,24) = 0.79, p = 0.54). The shoe weight does not affect the speed-dependent modulations in MoS when the weight is 0.3.

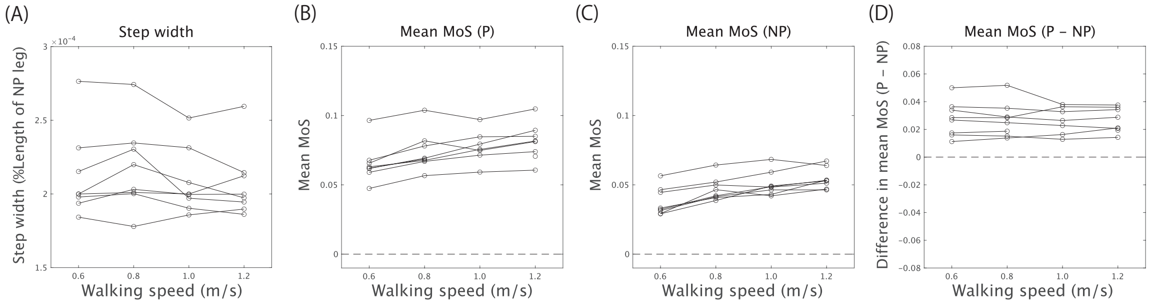

Fig. S8: Step width and MoS when shoe weight is 0.3(kg). (A): The relation between walking speed and step width. (B, C): The relation between walking speed and MoS in prosthetic (P) and non-prosthetic (NP) sides, respectively. (D): Asymmetry of MoS between prosthetic and non-prosthetic sides.

The case when shoe weight is 0.6kg

There was a significant main effect of velocity in the first harmonic in the mediolateral direction (F(4,28) = 27.6, $p = 2.3 \times 10^{-9}$), the second harmonic in the superoinferior direction (F(4,28) = 95.0, $p = 1.0 \times 10^{-15}$ [uncorrected]), and the fourth harmonic in the superoinferior direction (F(4,28) = 18.0, $p = 2.0 \times 10^{-7}$ [uncorrected]). There was no main effect of velocity on the first harmonic in the superoinferior direction (F(4,28) = 0.35, p = 0.84 [uncorrected]) and the third harmonic in the superoinferior direction (F(4,28) =0.73, p = 0.58 [uncorrected]). The shoe weight does not affect the speed-dependent modulations in amplitudes of each component when the weight is 0.6.

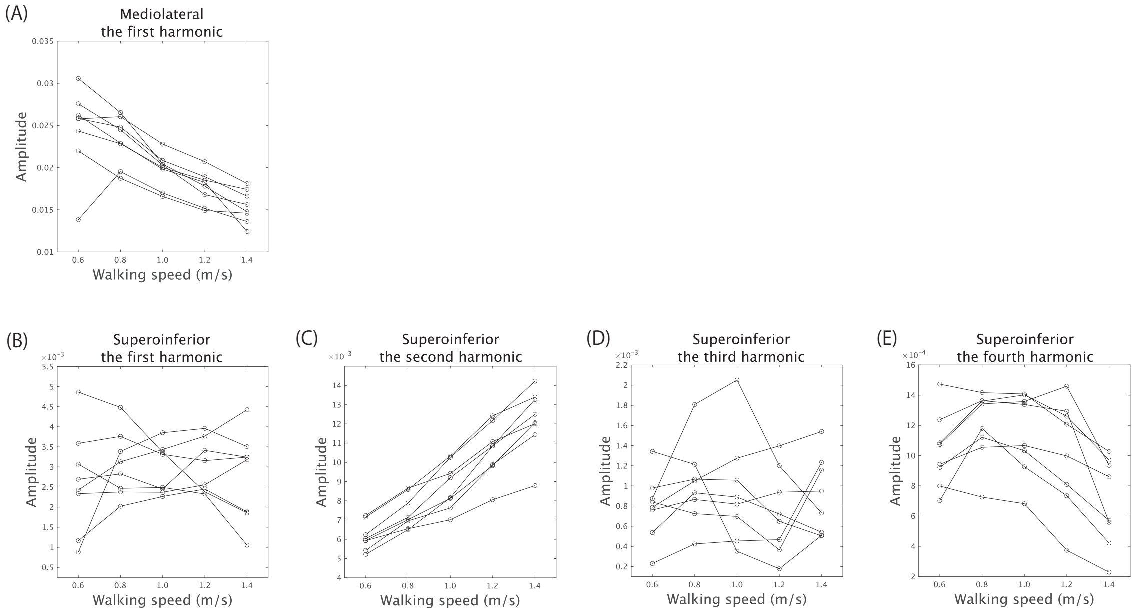

Fig. S9: Speed-dependent modulations of amplitudes in each component when shoe weight is 0.6(kg). (A): The modulations in the first harmonic in the mediolateral direction. (B-E): The speed-dependent modulations in the first, second, third, and fourth harmonics in the superoinferior direction.

In the analysis of MoS, there was a significant main effect of velocity (F(4,24) = 29.7, $p = 5.6 \times 10^{-9}$) and either prosthetic or non-prosthetic side (F(1,6) = 43.4, $p = 5.9 \times 10^{-4}$). There was no interaction between velocity and prosthetic/non-prosthetic side (F(4,24) = 0.81, p = 0.53). There was no main effect of velocity on the asymmetry of MoS (Asymmetry; F(4,24) = 0.81, p = 0.53). The shoe weight does not affect the speed-dependent modulations in MoS when the weight is 0.6.

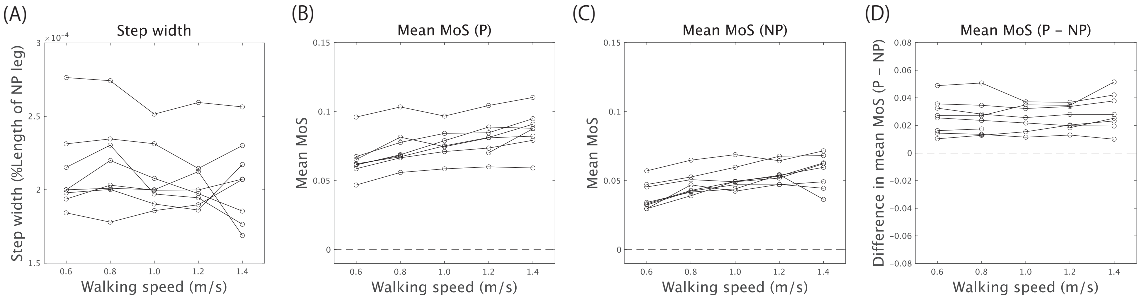

Fig. S10: Step width and MoS when shoe weight is 0.6(kg). (A): The relation between walking speed and step width. (B, C): The relation between walking speed and MoS in prosthetic (P) and non-prosthetic (NP) sides, respectively. (D): Asymmetry of MoS between prosthetic and non-prosthetic sides.

The analysis of one subject with prosthetic knee joint that utilizes hydraulic control

To examine the influence of the type of prosthetic knee, we compared the amplitude in each harmonic (Fig. S11) and MoS (Fig. S12) between the subjects with microprocessor-controlled knee (N = 7) and the subject with prosthetic knee joint that utilizes hydraulic control (N = 1). In the comparison between the group, the current study utilized two sample t-test with Bonferroni's correction.

There were significant differences between the group in the amplitude of the first harmonic component in the mediolateral direction while walking at 1.0, 1.2, and 1.4m/s (Fig. S11A, $p < 0.016$ [corrected]). In walking at 0.6 and 0.8m/s, there was no significant difference in the amplitude ($p > 0.12$ [corrected]). In the amplitude of the second harmonic in the superoinferior direction, the difference between the group was significant at 0.6 and 0.8m/s (Fig. S11C, $p < 0.013$ [corrected]). In walking at 1.0, 1.2, and 1.4m/s, there was no significant difference in the amplitude ($p > 0.81$ [uncorrected]). Although there was significant difference in the amplitude of the fourth harmonic while walking at 0.8, 1.0, 1.2, and 1.4m/s (Fig. S11E, $p < 0.014$ [corrected]), the difference is subtle due to its small magnitude as shown in Fig. 7. There was no significant difference in other cases ($p > 0.085$ [corrected]). Prosthetic knee joint that utilizes hydraulic control might affect the components majorly modulating the CoM trajectories (i.e., the first harmonic in the mediolateral direction and the second harmonic in the superoinferior direction) depending on walking speed (Fig. 7). Of note, the tendencies of the speed-dependent modulations were consistent between the two groups. Because these results were based on the analysis of single subject, future works are necessary to examine the influence of the type of prosthetic knee in detail.

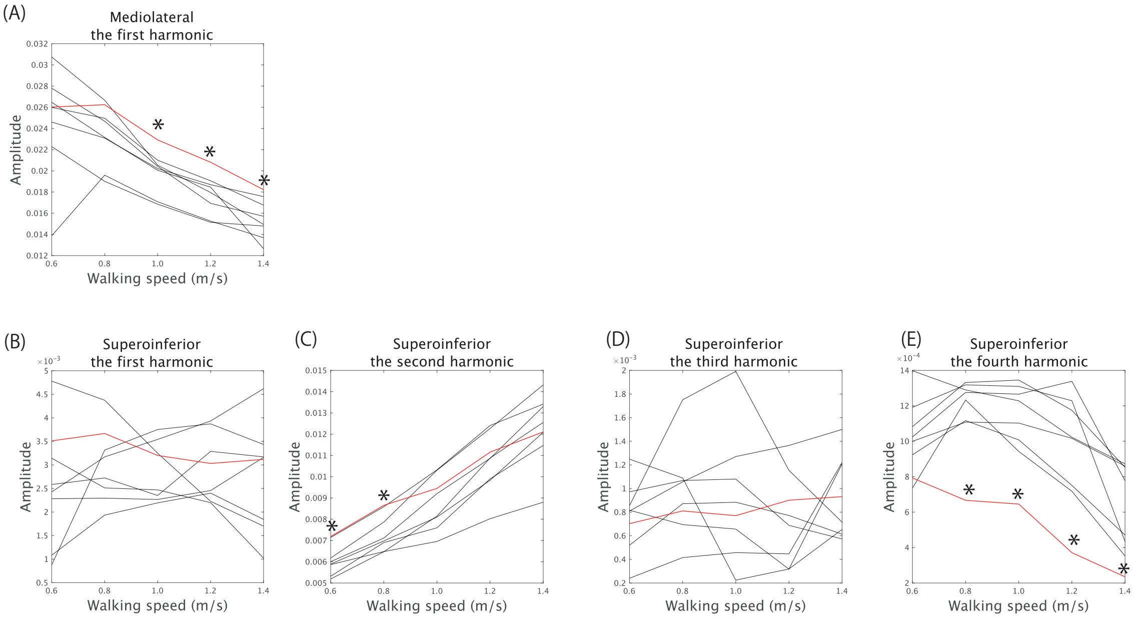

Fig. S11: Speed-dependent modulations of amplitudes in each component. Black lines and red line denote the modulations of subjects with microprocessor controlled knee (N = 7) and the subject with prosthetic knee joint that utilizes hydraulic control (N = 1), respectively. (A): The modulations in the the first harmonic in the mediolateral direction. (B-E): The speed-dependent modulations in the first, second, third, and fourth harmonics in the superoinferior direction.

There were significant differences in the MoS of prosthetic side between the group (Fig. S12B, $p < 0.04$ [corrected]). In contrast, there was no significant difference in the MoS of non-prosthetic side between the group (Fig. S12C, $p > 0.077$ [corrected]). In the difference of MoS between prosthetic and non-prosthetic side (Fig. S12D), there was significant difference while walking at 1.0, 1.2, and 1.4m/s ($p < 0.033$ [corrected], $p > 0.15$ while walking at 0.6 and 0.8m/s). Although prosthetic knee joint that utilizes hydraulic control might affect the MoS of prosthetic side, these results were based on the analysis of single subject. Future works are necessary to examine the influence of the type of prosthetic knee in detail. Of note, the tendencies of the speed-dependent modulations were consistent between the two groups.

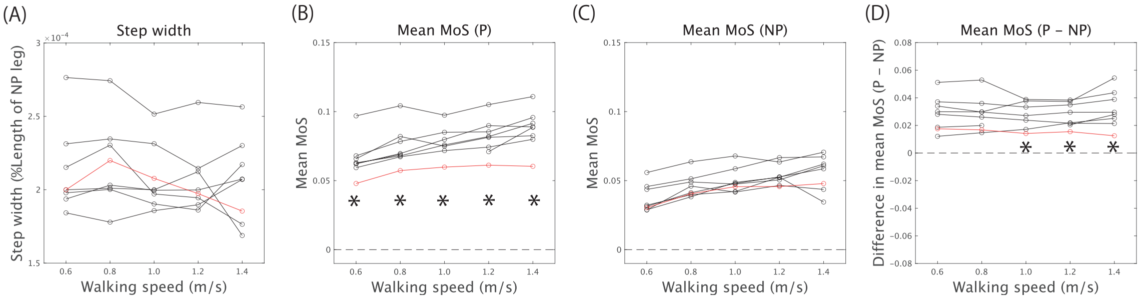

Fig. S12: Step width and MoS. Black lines and red line denote the modulations of subjects with microprocessor controlled knee (N = 7) and prosthetic knee joint that utilizes hydraulic control (N = 1), respectively. (A): The relation between walking speed and step width. (B, C): The relation between walking speed and MoS in prosthetic (P) and non-prosthetic (NP) sides, respectively. (D): Asymmetry of MoS between prosthetic and non-prosthetic sides.
